# Supplementary material for: Octave-spanning tunable infrared parametric oscillators in nanophotonics
Source: Sci Adv. 2023 Jul 26;9(30):eadf9711. doi: 10.1126/sciadv.adf9711 (PMC10371009; doi:10.1126/sciadv.adf9711)
Supplement: Supplementary file 1 — Figs. S1 and S2 [file sciadv.adf9711_sm.pdf]

Science Advances
AAAS

Supplementary Materials for

Octave-spanning tunable infrared parametric oscillators in nanophotonics

Luis Ledezma *et al.*

Corresponding author: Alireza Marandi, marandi@caltech.edu

Sci. Adv. **9**, eadf9711 (2023)
DOI: 10.1126/sciadv.adf9711

This PDF file includes:

Figs. S1 and S2

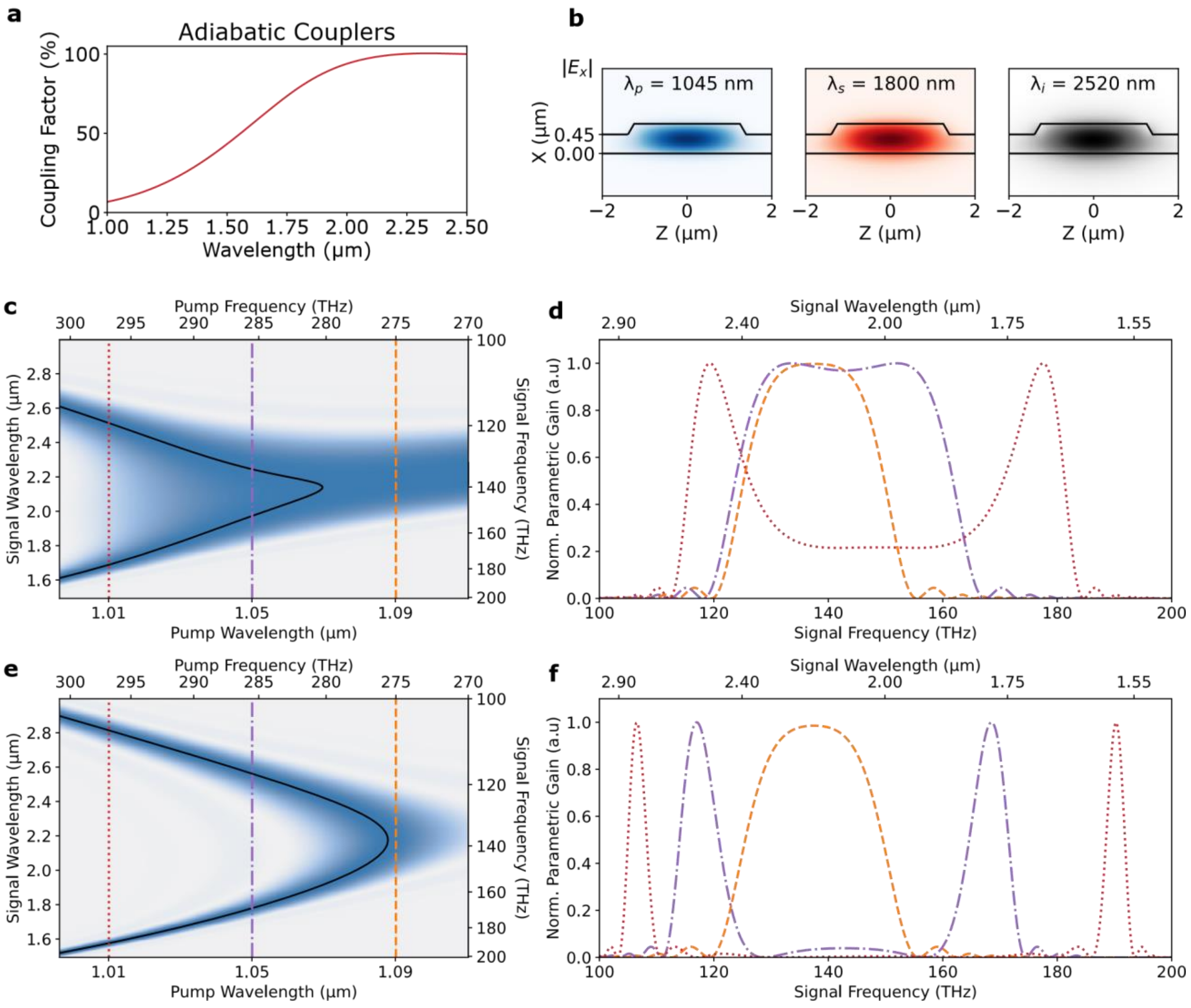

Fig. S1. Doubly-resonant OPO Design. a, Simulated coupling factor of adiabatic couplers used to create the OPO resonator. **b**, Simulated mode profiles for a representative set of pump, signal, and idler wavelengths, illustrating their similarities despite the large frequency difference leading to a substantial mode overlap over wide bandwidth. **c-f**, Two examples of OPOs with different geometries, showing the effect of dispersion engineering on the tuning curves. **c,d** Correspond to an OPO that could be pumped with femtosecond pulses for frequency comb generation in the mid-infrared. **e,f** Correspond to the OPOs described in the main text, exhibiting a smooth tuning characteristic. **d,f** Parametric gain as a function of signal (and idler) frequency for fixed pump wavelengths indicated by the vertical lines in **c,e**.

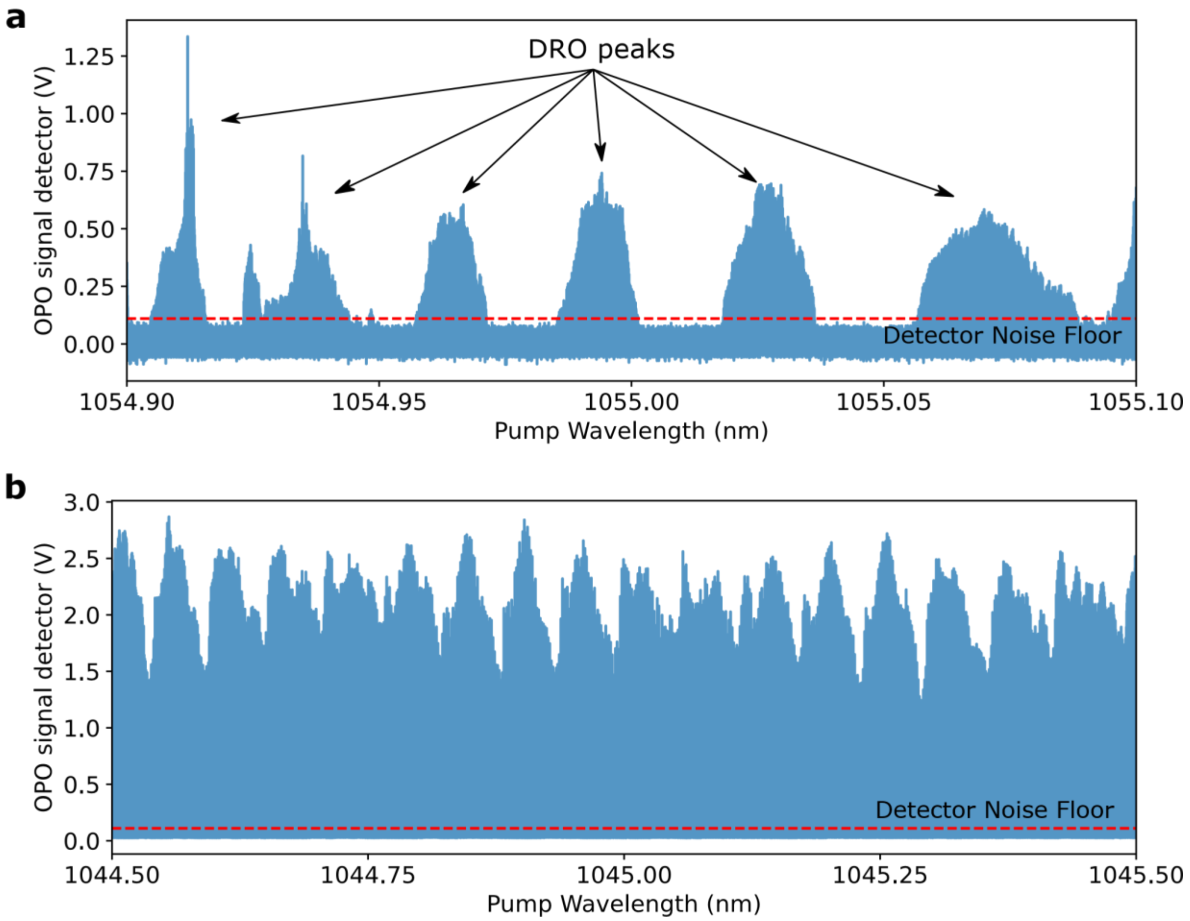

Fig. S2. Doubly-resonant and singly resonant regimes. a, Detected OPO signal for low pump power showing isolated oscillation peaks. This phenomenon, known as cluster effects in doubly-resonant OPOs (DROs), is due to the difference between the free spectral range at signal and idler wavelengths produced by waveguide dispersion. **b**, At larger pump powers the OPO oscillates for any pump wavelength. This is because away from a doubly-resonant cluster, the OPO operates closer to the singly resonant regime, with a strongly resonant idler and a weakly resonant signal that is free to adjust itself to a frequency $\omega_s = \omega_p - \omega_i$. In both, **a** and **b**, signal power variations are due to a combination of threshold variations and wavelength dependent pump laser power.
